# Impact of Whole-Body Cryotherapy on Pain, Sleep Quality, Functional Status, and Quality of Life in Multiple Sclerosis: A Comparative Study with Follow-Up

**DOI:** 10.3390/jpm15020046

**Published:** 2025-01-26

**Authors:** Ewa Zielińska-Nowak, Anna Lipert, Łukasz Kikowski, Elżbieta Miller

**Affiliations:** 1 Department of Neurological Rehabilitation, Medical University of Lodz, Milionowa 14, 90-001 Lodz, Poland; 2 Department of Preventive Medicine, Medical University of Lodz, 92-213 Lodz, Poland; anna.lipert@umed.lodz.pl; 3 Department of Allergology and Pulmonary Rehabilitation, Medical University of Lodz, 90-153 Lodz, Poland; lukasz.kikowski@creator.wroc.pl

**Keywords:** whole-body cryotherapy, cryostimulation, multiple sclerosis, pain, functional status, sleep quality

## Abstract

**Background/Objectives:** Whole-body cryotherapy (WBC) is widely recognized for its analgesic and anti-inflammatory effects. Despite growing interest in its therapeutic potential, the impact of WBC on functional performance, pain perception, sleep quality, and quality of life among individuals with multiple sclerosis (MS) remains underexplored. This study aimed to assess the effects of a 10-session WBC protocol on functional and psychological parameters in patients with MS and compare them with individuals without neurological disorders. **Methods:** A total of 73 participants divided into two groups, non-neurological individuals (non-MS, *n* = 43) and patients with MS (MS, *n* = 30), underwent 10 WBC sessions (−120 °C to −130 °C) over 2 weeks. Assessments included the Numerical Rating Scale (NRS), 30-Second Chair Stand Test (30CST), Timed Up and Go (TUG) test, and Pittsburgh Sleep Quality Index, with the WHOQOL-BREF conducted pre-treatment, post-treatment, and at a 10-day follow-up. **Results:** In the MS group, significant improvements were observed post-treatment in the NRS, 30CST, WHOQOL-1, and PSQI. However, only the CST and WHOQOL-3 maintained improvements during follow-up. In the non-MS group, statistically significant improvements were observed post-treatment across most parameters, except for the NRS and WHOQOL-3, with most effects diminishing by the follow-up. No deterioration in any assessed parameters was observed in either group. **Conclusions:** WBC demonstrates potential benefits for managing MS symptoms, particularly pain and sleep quality, with no observed deterioration in parameters and some effects emerging only during follow-ups, underscoring its safety and the need for further research on long-term outcomes.

## 1. Introduction

Multiple sclerosis (MS) is a chronic neuroinflammatory disease of the central nervous system, marked by demyelination, neuronal damage, and a range of disabling physical and cognitive impairments, which collectively impose a substantial socioeconomic and individual burden globally. MS manifests with a broad spectrum of symptoms, including fatigue, cognitive impairment, gait and mobility challenges, spasticity, bladder and bowel dysfunction, visual disturbances, sensory deficits, pain, and emotional disturbances, such as depression and anxiety [1], highlighting the complexity of its management and the need for individualized therapeutic approaches. Among the numerous symptoms of MS, pain has gained increasing recognition as a significant and multifaceted aspect of the disease, contrary to the earlier belief that MS is a painless condition [2]. Pain in MS, according to pathophysiological mechanisms, is classified into three categories: nociceptive pain (including pain from optic neuritis, musculoskeletal pain due to postural issues, lower back pain, migraines, tension-type headaches, and treatment-related pain); neuropathic pain (NP), presenting as persistent limb pain, Lhermitte’s sign, or trigeminal neuralgia; and mixed pain, such as painful tonic spasms and spasticity-related pain [2,3].

Another significant issue among individuals with MS is poor sleep quality, affecting over 50% of patients [4,5]. Sleep disturbances in this population can stem directly from the disease’s effects on the central nervous system, including demyelination and neuroinflammatory processes, or indirectly from related symptoms, such as pain, spasticity, fatigue, and depression. These disturbances often lead to worsened quality of life (QoL) and increased disease burden [5].

MS significantly affects both physical and psychological well-being, leading to reduced functionality and social participation. Research indicates that individuals with MS report consistently lower QoL compared to the general population, with factors such as fatigue, depression, and mobility impairments playing a prominent role in this decline [6]. The multifaceted impact of MS symptoms, including their cyclical interplay—where the worsening of one symptom, such as pain or fatigue, exacerbates others—underscores the importance of a holistic approach to treatment.

Symptomatic treatment remains a cornerstone of MS care, aiming to alleviate the disease burden and improve patients’ QoL. Over the past few decades, advancements in MS therapies have transformed the treatment landscape. Pharmacological approaches are effective in reducing relapse frequency and delaying disability progression but may not fully address all aspects of disease activity and their effectiveness is significantly limited in more progressive forms of MS, highlighting a need for complementary approaches in MS management [7]. Moreover, the side effects and limitations of long-term pharmacological treatments, including the risk of infections and systemic adverse effects, underscore the necessity of exploring complementary and non-pharmacological therapies [8].

Non-pharmacological approaches have gained increasing attention as essential components of comprehensive MS management. Rehabilitation, in particular, is increasingly recognized as a cornerstone of care, offering significant benefits for maintaining and improving functional status and fatigue, as well as enhancing quality of life in individuals with MS [9,10,11,12]. However, the growing complexity of MS symptoms highlights the need to explore and implement innovative solutions that address unmet therapeutic needs and complement existing treatments [13,14]. These strategies, which most frequently include physiotherapy, exercise programs, cognitive behavioral therapy, and lifestyle interventions, aim to address symptoms that remain unresponsive to pharmacological treatments [15].

In Poland, WBC has been widely adopted as a therapeutic intervention, with the national health fund reimbursing approximately 650,000 WBC sessions annually over the past decade [16]. Of these, an estimated 14,000 sessions were administered to individuals with MS, while the majority of these sessions were provided to patients with osteoarthritis (OA) and other musculoskeletal conditions.

In recent years, there has been a growing interest in the application of WBC among patients with MS, primarily due to its potential anti-inflammatory and antioxidative effects [17]. Studies suggest that WBC may reduce the pro-inflammatory cytokines, such as TNF-α and IL-1β, while increasing levels of anti-inflammatory cytokines like IL-10 [18]. These mechanisms are particularly significant in the context of MS, an autoimmune disease characterized by chronic inflammation, which plays a significant role in disease progression. This therapeutic approach holds promise as a complementary strategy to address inflammation-driven symptoms in MS management.

Although WBC has demonstrated benefits in conditions such as rheumatoid arthritis, fibromyalgia, ankylosing spondylitis, depression, anxiety, sleep disorders, post-exercise muscle soreness, post-COVID syndrome, and obesity [19], research on its effects on neurological conditions like MS exists but remains limited.

This study seeks to evaluate the impact of WBC on functional performance, pain perception, and sleep quality in patients with MS, comparing the outcomes with those in a non-neurological population.

To the best of our knowledge, this is the first study to evaluate the effects of WBC on pain and sleep quality in patients with MS. Additionally, it incorporates a follow-up phase to explore the longevity of these effects, offering a comprehensive perspective on the intervention’s impact. By investigating these effects, this study seeks to broaden the understanding of WBC’s role in MS management and highlight its potential as a complementary therapeutic tool for improving quality of life in diverse patient populations. In addition, correlation analyses were also performed to examine the interplay between various outcomes, offering a more holistic perspective on the potential interconnected effects of WBC.

## 2. Materials and Methods

### 2.1. Participants

**Figure 1:**
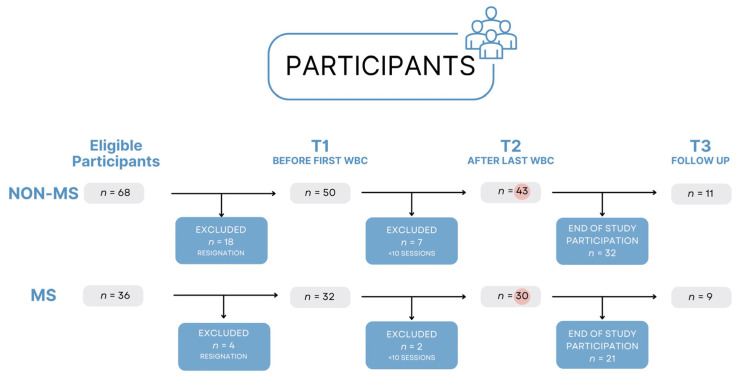
Participants flowchart. The orange circles indicate the number of participants who were assessed after completing 10 WBC sessions.

This study recruited participants from the Creator Rehabilitation Center in Łódź and divided them into two groups: those without neurological disorders (non-MS *n* = 43) and patients diagnosed with MS (MS *n* = 30) (Figure 1).

Patients from the MS group had been previously diagnosed with MS by a neurologist and are under regular care at a neurology outpatient clinic. The majority of MS patients (90%) were currently undergoing disease-modifying therapy while 10% were not. Regarding the type of MS, 76.7% of participants had relapsing–remitting multiple sclerosis (RRMS); 13.3% had primary progressive multiple sclerosis (PPMS); and in 10% of cases, the type of MS had not yet been determined. The mean baseline EDSS score for the MS group was 2.62 ± 1.43. In this group, 83% of participants had EDSS scores below 4, indicating no walking impairment, while only one participant required a walking aid (a cane), with an EDSS score of 6. The mean time since diagnosis was 9.88 ± 7.25 years (0.5; 30).

The non-MS group consisted of individuals recruited among patients referred for WBC due to OA, serving as a comparison group to highlight differences between those with and without neurological conditions. Inclusion criteria required patients to have a referral for WBC from a medical doctor, no contraindications for the therapy, an age range of 18 to 85 years, no neurological disorders in the WBC1 group and a confirmed diagnosis of MS in the WBC2 group, and written consent to participate.

Exclusion criteria included health conditions that contraindicate participation in WBC sessions, an EDSS score > 6, ongoing involvement in other physiotherapy treatments during the study period, and significant lifestyle or physical activity changes during or right before the WBC treatment period. Contraindications for WBC include untreated high blood pressure, recent heart attack within the past six months, decompensated cardiovascular or respiratory diseases, unstable angina, pacemakers, advanced peripheral artery occlusive disease (Fontaine Stages III and IV), history of deep-vein thrombosis, acute febrile diseases, severe anemia, seizure disorders, large-area bacterial or viral skin infections, and conditions such as cold allergy or severe wasting diseases. These conditions were thoroughly screened prior to this study to ensure patient safety [16].

The protocol and procedures were followed according to the Helsinki Declaration and were approved by the Ethics Committee of the Medical University of Lodz, Poland, RNN/97/23/KE, date of approval: 18 April 2023. The data were collected face to face.

### 2.2. Study Design

**Figure 2:**
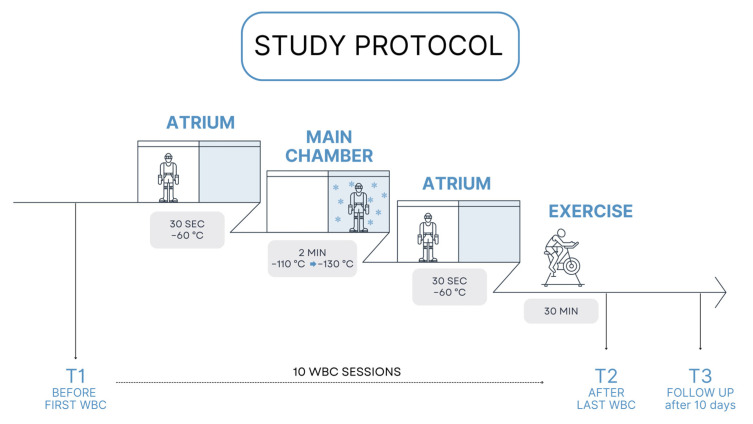
Study protocol.

Participants underwent a series of 10 consecutive WBC sessions, excluding weekends. The cryotherapy process took place in a specialized chamber with two sections. The procedure involved an initial adaptation phase of 30 s in an atrium at −60 °C, followed by 2 min in the main chamber. The temperature in the main chamber started at −120 °C on the first day and decreased by 2 °C every other day, reaching −130 °C by the last session. After the main chamber exposure, participants returned to the atrium for an additional 30 s. The whole procedure lasted for 3 min. The chamber was cooled using liquid nitrogen. To ensure safety, participants wore protective gear: gloves, knee-high socks, wooden clogs, a headband or hat for ear coverage, and a surgical mask. Each session was immediately followed by a 30 min supervised exercise routine, primarily on a cycle ergometer, to promote rewarming and enhance therapeutic benefits (Figure 2).

### 2.3. Assessment Protocol

Participants were evaluated at three points during this study: before the first cryotherapy session (T1), after completing the series of 10 sessions on the final treatment day (T2), and 10 days after the final session (T3). The clinical impact was assessed using the following scales and tests.

#### 2.3.1. Numerical Rating Scale (NRS) for Pain Assessment

The NRS is a widely utilized tool for subjective pain assessment, allowing patients to rate their pain intensity on a scale from 0 (no pain) to 10 (worst possible pain). Furthermore, its ease of administration—requiring patients to either verbalize or mark their pain level—ensures wide applicability [20]. Its simplicity makes it particularly suitable for clinical and research settings, including the evaluation of pain in patients with chronic and inflammatory conditions. As highlighted by Hawker et al., the NRS has demonstrated strong validity and reliability in diverse populations, where pain intensity plays a crucial role in determining functional status and treatment efficacy. Its sensitivity to change makes it an essential measure for assessing treatment outcomes and monitoring pain over time [21].

#### 2.3.2. The World Health Organization Quality of Life-BREF (WHOQOL-BREF)

The WHOQOL-BREF is a widely utilized instrument designed to assess quality of life across multiple domains (physical, psychological, social, and environmental) [22]. As highlighted in recent studies, this tool is particularly suitable for patients with chronic conditions, including neurological disorders like MS. It provides insights into how diseases impact day-to-day functioning and overall life satisfaction. Its user-friendly structure, consisting of 26 items rated on a 5-point Likert scale, allows for efficient data collection in both clinical and research settings [23].

#### 2.3.3. Pittsburgh Sleep Quality Index (PSQI)

The PSQI is a validated instrument for assessing subjective sleep quality, with a global score >5 typically indicating poor sleep [24]. It assesses seven components, including subjective sleep quality, latency, duration, habitual efficiency, disturbances, use of sleeping medication, and daytime dysfunction. The global PSQI score, ranging from 0 to 21, differentiates between “good” and “poor” sleepers [25]. However, no standardized cut-off score has been firmly established for individuals with MS [26]. Recent studies, including those on populations with chronic conditions, underscore the PSQI’s reliability and validity for evaluating sleep quality [26].

#### 2.3.4. 30-Second Chair Stand Test (30CST)

The 30CST is a widely used functional assessment to evaluate lower limb strength, balance, and fall risk, particularly useful in individuals with chronic neurological conditions, such as MS [27]. Participants sit in an armless chair approximately, with their feet flat on the ground and arms crossed over their chest. After receiving the start command, they transition from a seated to a standing position and back as many times as possible within 30 s. The total number of successful repetitions is recorded [28]. The 30CST has a good sensitivity to detect changes in functional performance over time [29], making it an excellent tool for tracking the effects of interventions like cryotherapy.

#### 2.3.5. Timed Up and Go (TUG)

The TUG test is a widely used and simple assessment tool for evaluating mobility, functional movement, and fall risk. In the TUG test, participants are required to rise from a seated position in a sturdy chair, walk three meters at a normal pace, turn around, return to the chair, and sit back down. Timing begins when the participant initiates movement and ends upon sitting [30]. This test provides insight into dynamic balance, gait speed, and the overall functional ability of individuals, making it a valuable measure for various populations, including those with neurological or musculoskeletal disorders [31].

### 2.4. Data Analysis

Statistical analyses were performed using Statistica version 13.1 software (StatSoft). The distribution of the variables was calculated using the Shapiro–Wilk test. The statistical analysis was performed using the Mann–Whitney U test for two independent variables or Kruskal–Wallis ANOVA for more than two independent variables. The effect size, measuring the differences between the results inside the groups and between groups, was determined by Cohen’s d; the value is defined as the difference between two means divided by a standard deviation for the data. Effect sizes were recorded as small (d = 0.2), medium (d = 0.5), and large (d = 0.8). Spearman’s correlation coefficient was used to assess the relationship between the results of the WHOQOL-BREF test results and quality of sleep. For all analyses, significant differences were accepted at the level of *p* < 0.05.

## 3. Results

In this research, we aimed to evaluate the impact of WBC on MS patients in comparison to a neurologically healthy population by focusing on the observed changes rather than the baseline results. However, it is important to note that the MS group exhibited poorer initial outcomes in the 30CST, PSQI, and WHOQOL-BREF Domains 1, 2, and 3 while the non-MS group did in the NRS, TUG test, and WHOQOL-BREF Domain 4. All of the results are listed in Table A1 and Table A2 in Appendix A.

The demographic and baseline characteristics of the study participants (n = 73) are detailed in Table 1, with a comparison between the non-MS group (n = 43) and MS group (n = 30) (Table 1).

The term “special diet” refers to both medically prescribed dietary interventions, such as gluten-free or low-fat diets, and individually chosen dietary practices adopted by participants, such as vegetarianism or other specific nutritional choices.

The data encompass factors such as gender distribution, education level, employment status, comorbidities, lifestyle habits, and prior exposure to cryotherapy, providing a thorough overview of the study population.

### 3.1. Changes in Test Results Before WBC, After 10 Sessions, and After 10 Days from the Last Session

In general, the changes in the results were greater and more often significant at T2 rather than T3. The results obtained at T3 were only slightly better than before 10 WBC sessions (Table 2).

#### 3.1.1. Numerical Rating Scale (NRS)

In the non-MS group, the changes in pain perception after cryotherapy were not statistically significant (Δ = −0.74, *p* = 0.081, d = 0.30). At T3 the results also showed no statistical significance but the effect size was moderate (Δ = −0.44, *p* = 0.515, d = 64). Conversely, in the MS group, the reduction in pain at T2 was statistically significant (Δ = −0.93, *p* = 0.031, d = 0.38). However, this effect did not persist at follow-up (Δ = −0.12, *p* = 1.00, d = 0.66).

#### 3.1.2. 30-Second Chair Stand Test (30CST)

Both groups experienced significant improvement in lower-limb strength and endurance at T2. In the non-MS group, the changes were statistically significant (Δ = 1.02, *p* = 0.020, d = 0.23). At T3, there was no significant change (Δ = 0.22, *p* = 0.475, d = 0.11). In MS patients, the improvement was greater and statistically significant with moderate effect size (Δ = 2.30, *p* = 0.001, d = 0.48) and a smaller, but still significant, improvement was maintained at T3 (Δ = 2.00, *p* = 0.038, d = 0.22).

#### 3.1.3. Timed Up and Go (TUG)

The non-MS group demonstrated significant improvement in functional mobility at T2 (Δ = −0.62, *p* = 0.002, d = 0.39) but no statistically significant changes were observed at T3 (Δ = −0.12, *p* = 0.155, d = 0.36). MS patients, neither in T2 (Δ = −0.30, *p* = 0.157, d = 0.12) nor T3 results (Δ = 0.07, *p* = 0.953, d = 0.03), showed statistical significance.

#### 3.1.4. WHOQOL-BREF Domains

- Somatic domain: Both groups improved at T2. In the non-MS group, the improvement was statistically significant and medium-sized (Δ = 5.07, *p* = 0.004, d = 0.41), with no significant effect at T3 (Δ = 1.19, *p* = 0.075, d = 0.42). Similarly, the MS group experienced significant improvements (Δ = 6.43, *p* = 0.001, d = 0.40) but these did not persist at T3 (Δ = 0, *p* = 0.726, d = 0.58);
- Psychological domain: Statistically significant improvements were observed in the non-MS group (Δ = 3.97, *p* = 0.010, d = 0.31). In the MS group, the changes were not significant at T2 (Δ = 0, *p* = 0.793, d = 0);
- Social domain: Improvements were not statistically significant for the non-MS group (Δ = 2.52, *p* = 0.265, d = 0.17) but were significant for the MS group, however, only at T3 (Δ = 8.33, *p* = 0.028, d = 0.42);
- Environmental domain: Statistically significant changes were observed in the non-MS group at T2 (Δ = 3.85, *p* = 0.025, d = 0.29) but these were not seen in MS patients at any stage.

#### 3.1.5. Pittsburgh Sleep Quality Index (PSQI)

The improvements in sleep quality were statistically significant in both groups at T2. For the non-MS group, the changes were modest (Δ = −0.83, *p* = 0.041, d = 0.28), with no significant improvement at T3 (Δ = −0.11, *p* = 0.674, d = 0.28). For MS patients, sleep quality improved significantly at T2 (Δ = −1.40, *p* = 0.011, d = 0.40) but no significant effects were maintained at T3 (Δ = −1.37, *p* = 0.183, d = 1.08).

**Figure 3:**
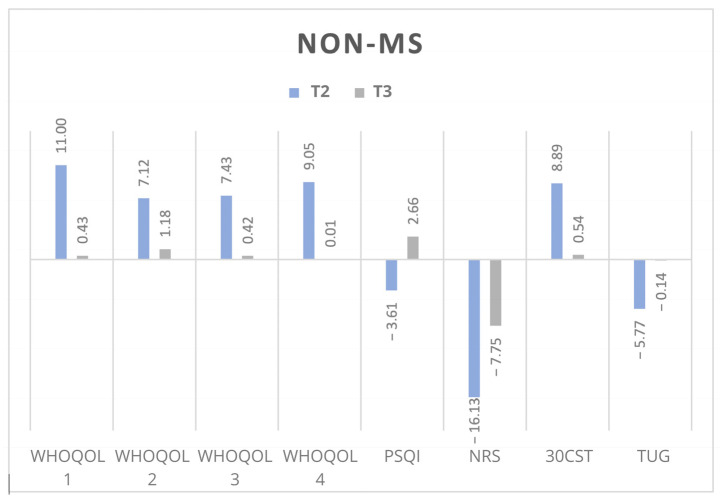
Percentage of change in the results of every test in non-MS participants. Abbreviations: WHOQOL: The World Health Organization Quality of Life-BREF; PSQI: Pittsburgh Sleep Quality Index; NRS: Numerical Rating Scale; 30CST: 30-Second Chair Stand Test; TUG: Timed Up and Go. T2: after last WBC; T3: at follow-up.

**Figure 4:**
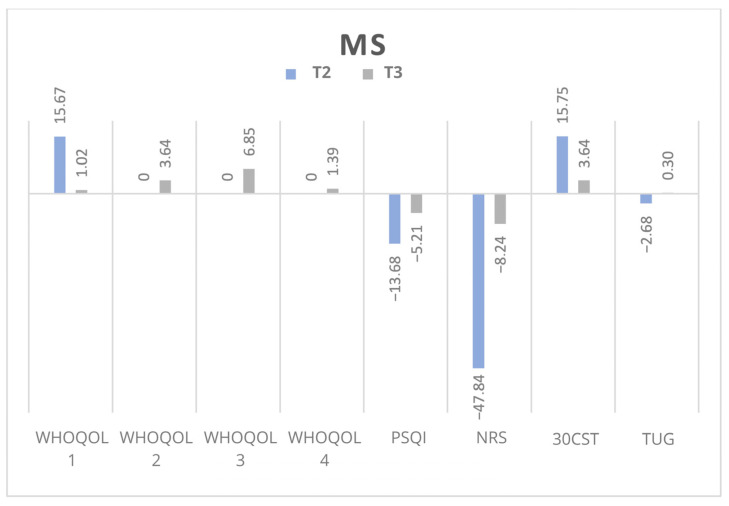
Percentage of change in the results of every test in MS participants. Abbreviations: WHOQOL: The World Health Organization Quality of Life-BREF; PSQI: Pittsburgh Sleep Quality Index; NRS: Numerical Rating Scale; 30CST: 30-Second Chair Stand Test; TUG: Timed Up and Go. T2: after last WBC; T3: at follow-up.

The most significant changes were observed at T2, with a notable decline in the persistence of these improvements at T3, particularly among MS patients. Figure 3 presents the percentages of the changes at T2 and T3 in the non-MS group and Figure 4 presents the same in the MS group.

### 3.2. Correlations Between the Quality of Life and Quality of Sleep After Cryotherapy

Spearman’s correlation test, as highlighted in Table 3, identified a relationship between the results of the WHOQOL-BREF questionnaire and the PSQI scores obtained at T2 in non-MS patients (Table 3).

In non-MS patients, a strong and positive relationship (r = 0.68; *p* < 0.05) was observed between the WHOQOL 3 and PSQI. Also, a statistically significant correlation was observed between the results of the WHOQOL 4 and PSQI obtained at follow-ups. In MS patients, no statistically significant correlations were observed.

### 3.3. Correlations Between the Number of Cryotherapy Sessions in the Past and Obtained Test Results

There was observed a relationship between the number of cryotherapy sessions in the past and the obtained results in the tests after cryotherapy in both, non-MS and MS patients, but in different time periods (Table 4).

In the MS group, there was mostly a negative and moderate relationship between the results 10 days after cryotherapy and the number of sessions in the past. However, the correlations changed into positive and moderate correlations in the WHOQOL-BREF domain 3 and 4 and in the 30CST follow-up. It means that in a longer period of time, more frequent cryotherapy sessions can positively influence some aspects of QOL and physical health.

In the non-MS group, only a positive and small correlation could be observed between the PSQI score and the number of sessions in the past but the results were not statistically significant. Similarly to the MS group, the correlations also changed at T3. A strong and positive statistically significant relationship was observed between the WHOQOL-BREF domain 1 results and the number of sessions in the past.

## 4. Discussion

We investigated the effects of WBC on pain, functional status, sleep quality, and quality of life in patients with MS compared to those without any neurological conditions (non-MS), including a follow-up assessment to evaluate the durability of these effects. While the benefits of WBC are well-documented in localized musculoskeletal conditions, such as OA, its impact on systemic and neurologically driven symptoms in MS remains less explored.

WBC has repeatedly demonstrated analgesic effects, not only among healthy athletes but also on various medical conditions [32,33,34], including OA. In a study by Chruściak et al., among OA patients, pain perception, its frequency, and the number of taken analgesic medications were reduced [35]. However, to the best of our knowledge, no studies have assessed the impact of a series of WBC sessions on pain in patients with MS. In this study, we aimed to examine the impact of cryostimulation on pain management in MS patients. In this study, we found that while WBC may alleviate pain in OA patients, its effects were more pronounced and statistically significant in individuals with MS. This reduction in pain may be attributed to the anti-inflammatory and neuromodulatory effects of cryotherapy, highlighting its potential as a valuable non-pharmacological intervention for managing chronic pain.

In patients with MS, sit-to-stand performance has been linked to lower-limb strength, spasticity, trunk control, and balance [29,36,37]. In our study, the 30CST was used to assess the impact of WBC on functional performance in both MS patients and individuals without any neurological conditions and the 30CST results highlighted notable differences between the non-MS and MS groups. In the non-MS group, the increase in repetitions at T2 was statistically significant, indicating a positive effect of cryotherapy on lower-limb strength. However, the magnitude of improvement was smaller compared to the MS group, where the change was also statistically significant but more noticeable.

The improvement observed in the 30CST for individuals with MS after WBC can be attributed to several key mechanisms. In MS patients, WBC appears to reduce fatigue and pain through its anti-inflammatory and neuromodulatory effects [37]. These changes may facilitate better neuromuscular coordination and muscle activity, allowing significant improvement in muscle strength and endurance tasks, such as repeated sit-to-stand movements. In contrast, the non-MS group also showed statistically significant improvement in the 30CST but the changes were less pronounced, possibly reflecting the more localized effects of WBC on nociceptive pain.

Interestingly, statistically significant improvements in the TUG test results were observed only in the non-MS group. This may reflect the nature of the task, which incorporates both sitting-to-standing movement and walking. For individuals with OA (non-MS group), pain relief and reduced stiffness in joints likely contributed to improved mobility. In contrast, MS patients showed improvements in TUG performance that were not statistically significant, potentially due to persistent neurological impairments, such as reduced balance and motor control, which are less immediately responsive to cryostimulation.

Although there are no studies using these specific tests to evaluate strength and functional state in MS patients undergoing WBC, existing research, such as the study by Radecka et al., has shown improvements in functional outcomes through other measures [37]. A series of 20 WBC sessions led to decreased fatigue, improved gait speed, and increased hand grip strength. These changes are attributed to adaptive modifications in bioelectrical muscle activity, as assessed through surface electromyography (sEMG) [38].

Additionally, a positive impact of WBC on functional status has been observed in studies using the Rivermead Motor Assessment (RMA). In the study conducted by Miller et al., MS patients were divided into two groups based on their levels of fatigue: low fatigue and high fatigue. Both groups demonstrated statistically significant improvements after 10 sessions of WBC, particularly in RMA Sections 1 (gross motor function) and 3 (arm movements) [37]. The improvements were especially pronounced in the high-fatigue group, highlighting the potential of cryotherapy to alleviate fatigue-related functional limitations.

Pawik et al. compared functional outcomes in MS patients divided into three groups: one undergoing WBC, another combining cryotherapy with structured physical exercise, and a third group participating only in a physical exercise program. While all groups showed improvements in functional status, the combined approach of cryotherapy and exercise yielded the most significant enhancements in the RMA [39]. This finding underscores the additive benefits of integrating physical activity with cryotherapy to maximize functional recovery and mobility in patients with MS.

Another common challenge faced by patients with MS is sleep disturbance. It is worth noting that our initial findings pointed to the poorer sleep quality in the MS group compared to the non-MS group, emphasizing the unique challenges faced by individuals with MS. While therapies, such as melatonin supplementation, cognitive behavioral therapy, chronotherapy, and non-invasive brain stimulation, as well as complementary methods, such as yoga, Tai Chi, and acupuncture, have been explored for improving sleep in MS patients [40], WBC could offer a novel and complementary approach, addressing sleep disturbances linked to other symptoms. Until now, the impact of WBC on sleep disturbances in individuals with MS has not been examined; however, its effects have been explored in other populations, highlighting its potential in this area. For example, research by Douzi et al. has shown that WBC can improve sleep quality in healthy individuals. A study conducted with physically active men reported that evening, post-workout, 3 min WBC sessions improved the subjective sleep quality and the morning form state; improved the objective sleep quality by reducing the number of movements during sleep; enhanced the pain relief; and improved parasympathetic nervous activity during the deep sleep (slow wave sleep) [41]. Given the prevalence of sleep disturbances among individuals with MS, these findings suggest that WBC may serve as a promising non-pharmacological intervention to alleviate sleep-related issues within this population.

While no studies to date have specifically examined the effects of WBC on sleep quality in MS patients, the improvements observed in our findings align with the broader benefits of WBC reported in other contexts. The statistically significant improvement in the MS group may be attributed to the complex interplay between these factors, particularly given the greater symptom burden in this population.

The lack of comparable studies underscores the novelty of our findings and highlights the potential of WBC as an adjunct therapy for addressing sleep disturbances in MS patients. Further research is required to explore these effects more comprehensively and to identify mechanisms that could explain the observed differences between the MS and non-MS groups.

The final parameter assessed in our study was the QoL (WHOQOL-BREF), which revealed notable differences between the non-MS and MS groups. Right after ten sessions of WBC (T2), we observed a greater improvement in QoL (WHOQOL-BREF) among non-MS participants, with statistically significant improvements in Domains 1 (somatic), 2 (psychological), and 4 (environmental) while Domain 3 (social) showed improvement without reaching statistical significance. At the same time (T2), among patients with MS, QoL improved significantly only in the somatic domain (Domain 1), with the other domains remaining unchanged. Notably, the somatic domain in MS participants demonstrated a greater degree of improvement compared to non-MS participants. Interestingly, during the T3 examination, the improvements observed in the non-MS group began to return toward baseline values. In contrast, among MS patients, there was a trend toward improvement in the remaining domains, with a notable and statistically significant enhancement (*p* = 0.028) in Domain 3 (social). These findings may suggest that the time frame between the intervention and assessment might have been insufficient to capture broader changes or that patients with MS may require more than functional improvements to observe changes in other aspects of life beyond the somatic domain. Moreover, while the effects of WBC on QOL may diminish over time in non-MS participants, the benefits for MS patients could emerge more gradually, particularly in domains beyond the somatic. This warrants further investigation into the mechanisms driving these differences and the potential long-term benefits of WBC in MS.

To the best of our knowledge, this is the first study to evaluate the effects of WBC on QoL in patients with MS using the WHOQOL-BREF questionnaire. Previous research, such as that of Szczepańska-Gieracha et al. [42], has demonstrated significant improvements in the WHOQOL-BREF after 10 sessions of WBC in patients with spinal pain syndromes and peripheral joint diseases. Vitenet et al. [43] documented improvements in the QoL of fibromyalgia patients measured with the Medical Outcome Study Short Form-36 (MOS SF-36), particularly in terms of health-related QoL, further reinforcing the potential of WBC to enhance QoL in diverse clinical populations. These findings underscore the need for further research to explore the mechanisms underlying these effects and to establish standardized approaches to assess and optimize WBC protocols in different patient populations.

It is important to consider that most symptoms in MS are interconnected, where the worsening of one aspect can significantly impact others. For instance, pain and spasticity may exacerbate fatigue while cognitive impairment and depression can further diminish overall quality of life. This relationship highlights the necessity for a holistic approach to managing MS symptoms [44].

A noteworthy and valuable trend emerged in our study in the relationship between the number of previous cryotherapy sessions and outcomes, particularly during the follow-up period. While no significant correlations were observed immediately post-intervention, the follow-up analysis revealed a positive influence of past sessions on several parameters in both the MS and non-MS groups. These observations suggest that the frequency of cryotherapy sessions could play a critical role in amplifying and sustaining therapeutic effects, particularly over extended periods.

Importantly, none of the assessed parameters, in either group, demonstrated any deterioration throughout the study period. This finding suggests that WBC is not only a potentially effective intervention but also a safe one, with no adverse impact on the physical, functional, or psychological state of participants. Such results underline the stability and tolerability of cryotherapy, making it a promising adjunctive therapy for improving various aspects of health without posing additional risks.

Regardless of the promising findings of this study, several limitations should be considered when interpreting the results. This study focused on a specific subgroup of patients with low to moderate levels of disability and included predominantly women, which could limit the generalizability of the findings. As MS is a highly heterogeneous condition, characterized by a wide range of symptoms, further research is needed to explore the effects of WBC on other dimensions of the disease that were not captured in this study. Moreover, the assessment was conducted using a limited set of tools and more comprehensive evaluations—particularly for complex aspects like sleep quality—would require the integration of additional, multidimensional tools to provide a fuller understanding. We also acknowledge the lack of complete investigator blinding as a limitation of this study; however, this constraint was inherent to this study’s design, as the groups were assessed in separate cohorts over the course of a year. While investigators were unaware of participants’ personal information, knowledge of patient conditions was necessary to ensure accurate data collection and patient safety. Moreover, certain tools were exclusively applicable to the MS group.

An additional limitation of this study is the relatively small number of participants who completed the T3 follow-up assessment, which may have affected the statistical power of detecting long-term effects. Furthermore, the short period between T2 and T3 might not have been sufficient to fully capture the durability of certain benefits, warranting future studies with extended follow-up periods to better understand the long-term impact of WBC. Despite these limitations, this study demonstrates that WBC is a well-tolerated therapy, with no adverse events or complaints reported among the participants.

## 5. Conclusions

The findings of this study suggest that WBC may offer meaningful benefits in managing symptoms associated with MS, particularly in areas such as pain relief and sleep quality. These results indicate that the benefits of WBC could be especially evident when applied in a cyclical manner, highlighting the potential value of repeat interventions. Notably, while some effects of WBC were observed immediately after therapy, others appeared over time, as demonstrated by the delayed improvements in certain WHOQOL-BREF domains among MS patients during follow-up sessions. Importantly, no deterioration in the assessed parameters was observed in either group, which aligns with existing evidence supporting the safety of WBC as a non-invasive therapeutic approach. These observations underline the need for further exploration into the long-term effects of WBC and its role in symptom management for MS. Future research could benefit from longer follow-up periods and a broader range of assessments to better understand both the delayed and transient effects of WBC, as well as its potential to address the unique needs of different patient populations.

## Figures and Tables

**Table 1:** The demographic and baseline characteristics of the study participants.

	All (*n* = 73)	NON-MS (*n* = 43)	MS (*n* = 30)
**Mean ± SD (95% CI)**			
**Age** (yr)	54.36 ± 14.31 (50.99; 57.72)	61.59 ± 9.52 * (58.63; 64.56)	44.23 ± 13.83 (39.07; 49.40)
**Weight** (kg)	69.66 ± 12.84 (66.66; 72.65)	69.07 ± 12.26 (65.30; 72.84)	70.50 ± 13.79 (65.35; 75.65)
**Height** (cm)	166.92 ± 8.56 (164.92; 168.91)	164.81 ± 7.78 * (162.42; 167.21)	169.93 ± 8.83 (166.63; 173.23)
**Previous WBC sessions**	5.32 ± 6.93 (3.69; 6.95)	5.64 ± 5.04 * (4.07; 7.21)	4.87 ± 9.02 (1.50; 8.24)
**N (%)**			
**Gender**			
Females	62 (83.78)	39 (88.64)	23 (76.67)
Males	11 (14.86)	4 (9.09)	7 (23.33)
**Education**			
Primary education	2 (2.70)	2 (4.54)	0
Secondary education	28 (37.84)	21 (47.73) *	7 (23.33)
Higher education	43 (58.11)	20 (45.45) *	23 (76.67)
**Working status**			
employed	45 (60.81)	22 (50.00) *	23 (76.67)
unemployed	1 (1.35)	0	1 (3.33)
retiree	23 (31.08)	19 (43.18) *	4 (13.33)
Disability pensioner	4 (5.40)	2 (4.54)	2 (6.67)
**Comorbidities**			
No	36 (48.65)	18 (40.91)	18 (60.00)
Yes	37 (51.35)	25 (59.09)	12 (40.00)
**Smoking**			
No	61 (82.43)	36 (81.82)	25 (83.33)
Yes	12 (16.22)	7 (18.18)	5 (16.67)
**Special diet**			
No	64 (86.49)	34 (77.27) *	30 (100.00)
Yes	9 (12.16)	9 (20.45)	0
**Physical activity**			
>2 times per week	33 (44.59)	20 (45.45)	13 (43.33)
2 times per week	15 (20.27)	11 (25.00)	4 (13.33)
1 time per week	13 (17.57)	7 (15.91)	6 (20.00)
1 time per month	3 (4.05)	1 (2.27)	2 (6.67)
No physical activity	9 (12.16)	4 (9.09)	5 (16.67)
**WBC in the past**			
No	18 (24.32)	3 (6.82) *	15 (50.00)
Yes	55 (74.32)	40 (90.91) *	15 (50.00)

Statistical significance when * *p* < 0.05.

**Table 2:** Changes in test results before WBC, after 10 sessions, and after 10 days from the last session.

	Non-MS (*n* = 43)						MS (*n* = 30)					
	Δ Changes	d	*p*	Δ Follow-Up	d	*p*	Δ Changes	d	*p*	Δ Follow-Up	d	*p*
**NRS**	−0.74 ± 2.65 (−1.56; 0.07)	0.30	0.081	−0.44 ± 2.65 (−2.48; 1.59)	0.64	0.515	−0.93 ± 2.16 (−1.74; −0.12)	0.38	0.031 *	−0.12 ± 2.53 (−2.24; 1.99)	0.66	1.00
**30CST**	1.02 ± 2.95 (0.11; 1.93)	0.23	0.020 *	−0.22 ± 3.60 (−2.48; 1.59)	0.11	0.475	2.30 ± 2.89 (1.22; 3.38)	0.48	0.001 *	2.00 ± 1.85 (0.45; 3.55)	0.22	0.038*
**TUG**	-0.62 ± 1.52 (0.12; 1.93)	0.39	0.002 *	−0.12 ± 1.37 (−1.18; 0.93)	0.36	0.155	−0.30 ± 1.12 (−0.72; 0.12)	0.12	0.157	0.07 ± 0.56 (−0.39; 0.54)	0.03	0.953
**WHOQOL 1**	5.07 ± 11.19 (1.62; 8.51)	0.41	0.004 *	1.19 ± 3.09 (−1.19; 3.57)	0.42	0.075	6.43 ± 9.21 (2.99; 9.87)	0.40	0.001 *	0 ± 9.73 (−8.14; 8.14)	0.58	0.726
**WHOQOL 2**	3.97 ± 9.36 (1.09; 6.85)	0.31	0.010 *	3.70 ± 4.86 (−0.03; 7.44)	0.40	0.093	0 ± 0.21 (−0.11; 0.11)	0	0.793	5.73 ± 9.94 (−2.58; 14.04)	0.35	0.116
**WHOQOL 3**	2.52 ± 13.79 (-1.72; 6.76)	0.17	0.265	0 ± 8.33 (−6.40; 6.40)	0	0.893	0 ± 0.03 (−0.01; 0.01)	0	0.991	8.33 ± 10.91 (−0.79; 17.45)	0.42	0.028*
**WHOQOL 4**	3.85 ± 9.76 (0.85; 6.85)	0.29	0.025 *	−0.35 ± 5.94 (−4.91; 4.22)	0.22	0.953	0 ± 0.20 (−0.10; 0.10)	0	0.699	1.56 ± 6.25 (−3.66; 6.79)	0.21	0.286
**PSQI**	-0.83 ± 2.70 (-1.67; -0.01)	0.28	0.041 *	−0.11 ± 2.03 (−1.67; 1.45)	0.28	0.674	−1.40 ± 2.76 (−2.43; -0.37)	0.40	0.011 *	−1.37 ± 3.33 (−4.16; 1.41)	1.08	0.183

Statistical significance when * *p* < 0.05.Abbreviations: NRS: Numerical Rating Scale; 30CST: 30-Second Chair Stand Test; TUG: Timed Up and Go; WHOQOL: The World Health Organization Quality of Life-BREF; PSQI: Pittsburgh Sleep Quality Inde.

**Table 3:** Correlations between the QoL and quality of sleep after 10 WBC sessions.

PSQI		QOL 1	QOL 2	QOL 3	QOL 4
non-MS	T2	−0.02	0.33	0.54	0.83
	T3	−0.13	0.22	0.68	0.11
MS	T2	0.12	−0.18	0.04	0.03
	T3	−0.37	−0.50	0.02	−0.02

Abbreviations: PSQI: Pittsburgh Sleep Quality Index; QOL: Quality of Life.

**Table 4:** Correlations between the number of cryotherapy sessions in the past and obtained test results.

Number of WBC Treatment Sessions in the Past		QOL 1	QOL 2	QOL 3	QOL 4	PSQI	NRS	30CST	TUG
non-MS	T2	0.12	−0.10	−0.09	0.07	0.36	−0.22	−0.25	−0.36
	T3	0.84	0.68	0.04	−0.02	0.59	0.62	0.02	0.30
MS	T2	−0.58	−0.25	−0.58	−0.51	0.41	−0.66	0.08	−0.58
	T3	0.01	0.21	0.33	0.60	−0.37	−0.27	0.22	−0.63

Abbreviations: QOL 1: WHOQOL-BREF domain 1; QOL 2: WHOQOL-BREF domain 2; QOL 3: WHOQOL-BREF domain 3; QOL 4: WHOQOL-BREF domain 4.

## Data Availability

The original contributions presented in this study are included in the article. Further inquiries can be directed to the corresponding authors.

## Appendix A

**Table A1:** Outcomes at Baseline, Post-Intervention, and Follow-Up in the non-MS group.

Non-MS	T1		T2		T3	
	Mean ± SD	Median (±95% CI)	Mean ± SD	Median (±95% CI)	Mean ± SD	Median (±95% CI)
NRS	4.23 ± 2.71	4.00 (3.40; 5.06)	3.49 ± 2.33 ^M^	3.00 (2.77; 4.21)	2.89 ± 1.67 ^M^	3.00 (1.58; 3.69)
30CST	16.00 ± 4.61	16.00 (14.58; 17.42)	17.02 ± 4.56 *^,S^	16.00 (15.62; 18.43)	16.45 ± 3.17 ^S^	16.00 (14.32; 18.59)
TUG	8.72 ± 1.62	8.56 (8.22; 9.21)	8.10 ± 1.56 **^,M^	7.83 (7.62; 8.58)	8.15 ± 1.56 ^M^	7.75 (7.10; 9.20)
WHOQOL 1	63.87 ± 13.55	64.29 (59.70; 68.04)	69.94 ± 11.44 **^,M^	67.85 (65.41; 72.45)	69.16 ± 8.64 ^M^	71.43 (63.35; 74.96)
WHOQOL 2	69.57 ± 13.16	70.83 (65.52; 73.62)	73.55 ± 12.92 **^,M^	70.83 (69.57; 77.52)	74.62 ± 11.7 ^M^	79.17 (66.76; 82.48)
WHOQOL 3	71.90 ± 16.27	75.00 (66.89; 76.90)	74.42 ± 14.01^S^	75.00 (70.10; 78.73)	71.97 ± 9.33 ^S^	75.00 (65.70; 78.24)
WHOQOL 4	64.32 ± 14.05	65.63 (59.99; 68.64)	68.17 ± 12.37 *^,M^	71.88 (64.36; 71.97)	67.33 ± 13.43 ^S^	65.63 (58.31; 76.35)
PSQI	6.07 ± 3.41	5.00 (5.02; 7.12)	5.23 ± 2.5 *^,M^	5.00 (4.46; 6.00)	5.18 ± 1.89 ^S^	5.00 (3.91; 6.45)

* *p* < 0.05, ** *p* < 0.01; The letters M, and S indicate moderate, and small effect sizes, respectively. Abbreviations: NRS: Numerical Rating Scale; 30CST: 30-Second Chair Stand Test; TUG: Timed Up and Go; WHOQOL: The World Health Organization Quality of Life-BREF; PSQI: Pittsburgh Sleep Quality Index.

**Table A2:** Outcomes at Baseline, Post-Intervention, and Follow-Up in the MS group.

MS	T1		T2		T3	
	Mean ± SD	Median (±95% CI)	Mean ± SD	Median (±95% CI)	Mean ± SD	Median (±95% CI)
NRS	2.6 ± 2.85	1.5 (1.54; 3.66)	3.49 ± 2.15 *^,S^	0.5 (0.86; 2.47)	0.89 ± 1.83 ^M^	0 (−0.52; 2.30)
30CST	14.7 ± 3.98	14.00 (13.21; 16.19)	17.00 ± 5.55 ***^,M^	15.5 (14.93; 19.07)	15.56 ± 3.71*	16.00 (12.70; 18.41)
TUG	8.42 ± 2.58	7.96 (7.46; 9.39)	8.17 ± 2.51 ^S^	7.82 (7.18; 9.06)	8.33 ± 3.27 ^S^	8.20 (5.82; 10.84)
WHOQOL 1	55.83 ± 16.95	55.36 (49.50; 62.16)	62.26 ± 15.77 ***^,M^	66.07 (56.37; 68.15)	65.08 ± 13.81 ^M^	67.85 (54.47; 75.69)
WHOQOL 2	63.47 ± 13.83	64.58 (58.30; 68.63)	63.47 ± 13.83	64.58 (58.31; 68.63)	68.06 ± 12.5 ^S^	62.5 (58.45; 77.66)
WHOQOL 3	65.00 ± 20.58	66.67 (57.32; 72.68)	65.00 ± 20.58	66.67 (57.32; 72.68)	73.15 ± 18.53 *^,M^	75.00 (58.90; 87.39)
WHOQOL 4	67.19 ± 14.33	65.63 (61.84; 72.54)	67.19 ± 14.33	65.63 (61.84; 72.54)	70.14 ± 14.41 ^S^	68.75 (59.06; 81.21)
PSQI	7.8 ± 3.6	7.5 (6.45; 9.16)	6.4 ± 3.58 **^,M^	6.00 (5.06; 7.73)	4.22 ± 2.49 ^S^	5.00 (2.31; 6.13)

* *p* < 0.05, ** *p* < 0.01, *** *p* < 0.001; The letters M, and S indicate moderate, and small effect sizes, respectively. Abbreviations: NRS: Numerical Rating Scale; 30CST: 30-Second Chair Stand Test; TUG: Timed Up and Go; WHOQOL: The World Health Organization Quality of Life-BREF; PSQI: Pittsburgh Sleep Quality Index.
